# Testing the environmental Kuznets curve hypothesis in terms of ecological footprint and CO_2_ emissions through energy diversification for Turkey

**DOI:** 10.1007/s11356-023-26278-w

**Published:** 2023-03-24

**Authors:** Hakan Acaroğlu, Hatice Melissa Kartal, Fausto Pedro García Márquez

**Affiliations:** 1 Department of Economics, Faculty of Economics and Administrative Sciences, Eskisehir Osmangazi University, 26480 Eskisehir, Turkey; 2 Institute of Social Sciences, Department of Economics, Yildiz Technical University, Istanbul, Turkey; 3 Ingenium Research Group, University of Castilla-La Mancha, Ciudad Real, Spain

**Keywords:** The EKC hypothesis, Ecological footprint, CO_2_ emissions, Economic growth, Renewable energy consumption, Energy diversification, ARDL bounds test

## Abstract

This research work analyzes the relationship between environmental degradation, economic growth, trade openness, primary energy consumption, coal consumption, and hydroelectricity consumption in Turkey from 1971 to 2015 using the autoregressive distributed lag (ARDL) time series approach through the hypothesis of the environmental Kuznets curve (EKC). Carbon dioxide (CO_2_) emissions and ecological footprint are both used as indicators of environmental degradation, which employs six different models. According to the results found in this study, while trade openness increases CO_2_ emissions, it decreases ecological footprint in the long-run. Coal consumption raises both CO_2_ emissions and ecological footprint. While hydroelectric energy reduces CO_2_ emissions, it has no effect on the environment. The results demonstrate that the EKC hypothesis is correct for both CO_2_ emissions and Turkey’s ecological footprint. The threshold points are investigated as $18,704, $16,361, and $13,571 in models, where CO_2_ emissions are the dependent variable. In models where the ecological footprint is the dependent variable, the investigated threshold points of $11,824, $11,821, and $15,476 are higher than the gross domestic product (GDP) per capita during the analysis periods. Furthermore, the findings highlight the importance of renewable energy use in reducing environmental degradation and coal use in increasing environmental degradation. These findings can shed light on the importance of transition to renewable energy sources (i.e., hydroelectricity consumption), from fossil fuels (i.e., coal consumption), related to future planning in energy diversification for Turkey.

## Introduction

Energy consumption has a significant impact on a country’s economic growth, while fossil fuels are still the most widely used energy source on a global scale (IEA 2022). Along with population and economic growth, however, the use of fossil fuels is one of the primary factors contributing to an increase in ecological footprint (*EF*) and CO_2_ emissions (Liu et al. 2022). CO_2_ emissions in 2020 increased by 149% over the pre-industrial revolution (1750) level (Organization 2021). While *EF* did not exceed the biocapacity until 1970, the world’s biological capacity has been exceeded by at least 56% in the twenty-first century (WWF 2020). These indicators show the direct cause of global warming and climate change, which is a serious threat to humanity, and their negative effects have been known scientifically for the last two decades. In this context, the number of interdisciplinary works combining the environment, economics, and statistics is growing, which is critical for finding solutions to these problems.

To this end, the study of testing the environmental Kuznets curve (EKC) hypothesis (i.e., the relationship between environmental degradation, economic growth, and energy consumption is generally considered within the scope of the EKC hypothesis) in terms of *EF*, and CO_2_ emissions through energy diversification for a developing country is necessary and important, and explains an investigation of the transition strategy from fossil fuel use to renewable energy use in the Turkish economy. With this motivation, the study is being done through both renewable energy consumption (REC) and non-renewable energy consumption (NREC) for sustainable economic growth, and proposes the solutions in the context of Turkey’s deteriorating environmental quality. Furthermore, it includes the observation of trade openness data (i.e., Turkey is a small open economy) for obtaining statistically more accurate results and can serve as an economic model for other less developed, developing, and developed countries attempting to mitigate global warming.

**Fig. 1:**
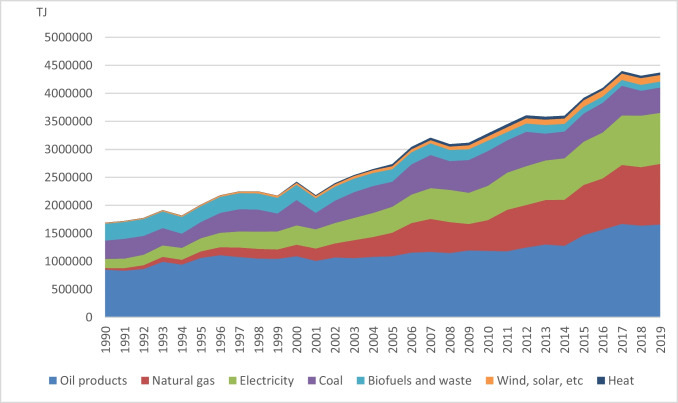
Evolution of the total energy consumption by source over the years in Turkey Source: Adapted from the Internal Energy Agency (IEA) (Agency 2021)

According to statistical information, the energy sector makes the biggest contribution to the formation of CO_2_ emissions in Turkey (TÜİK 2022a). Turkey depends on fossil fuel consumption for its energy resources, and in 2019, fossil fuels accounted for 83% of total primary energy consumption (Agency 2021). The development of total energy consumption over the years by source in Turkey is shown in Fig. 1, adapted from Agency (2021). According to Fig. 1, oil, natural gas, and coal are the most consumed energy sources. However, Turkey is highly dependent on imports (i.e., 99% and 93% of natural gas and oil, respectively, and 58% of coal demand is imported) in its energy use (Agency 2021), and this causes a current account deficit (Bulut and Muratoglu 2018). Turkey’s preference for fossil fuel consumption and its dependence on energy imports cause negative economic and environmental consequences. Nonetheless, these issues have increased enthusiasm for renewable energy production and consumption in recent years (Yurtkuran 2021). Therefore, this study uses primary energy consumption and coal consumption data for NREC and hydroelectricity consumption data for REC in its economic model.

The connection between REC and hydroelectricity consumption in the chosen economic model can be explained with necessary terminology as follows: Hydropower is a type of renewable energy generation, and hydroelectricity is a safe and cheap form of hydropower that produces electricity from the gravitational force of falling water (Bandyopadhyay et al. 2022). Furthermore, hydropower is the most widely used renewable energy source in Turkey as well as in the world (Ritchie and Rosado 2022), and Turkey provided approximately 29.2% of total electricity generation from hydropower in 2019; nonetheless, the availability of hydropower depends on hydrological conditions and can vary significantly (i.e., the sudden drop in hydropower production of Turkey in 2014 was due to a drought that year) (IEA 2021). As a result, it might have a correlation with economic growth, and despite the high initial costs of the projects, the consumption of hydroelectricity is expected to reduce environmental degradation through *EF* and CO_2_ emissions (Jahanger et al. 2022). Although there is not a consensus on the impacts of hydroelectricity consumption in developing countries, this study’s findings can be evidence for investigators and decision-makers through Turkey. Because the long-run trend for hydropower is increasing (i.e., with electricity generation from hydropower nearly tripling since 2000 in Turkey), and there is potential for further growth in the country (IEA 2021).

In addition, coal has an important place in Turkey (i.e., coal provided 38% of energy production of the country in 2019), in terms of reducing energy imports. For this reason, policies are aimed at increasing local coal production and consumption in Turkey (IEA 2021). However, coal consumption has a causality relationship with environmental degradation. The high proportion of coal in Turkey’s energy system (for instance, total coal consumption increased by 39% in a decade, from 29.4 Mtoe in 2008 to 40.8 Mtoe in 2018) is a major cause of greenhouse gas emissions in the country, and coal combustion accounted for 43% of energy-related CO_2_ emissions in 2018, and coal-related emissions have increased by nearly 32% in the last decade (IEA 2021). For this reason, the effect of coal consumption on environmental degradation in Turkey should be investigated.

This study addresses solutions of the aforementioned issues in the context of the EKC hypothesis and discusses whether the EKC hypothesis is valid across both CO_2_ emissions and EF. While testing this hypothesis, it is known that the Turkish government adopted a comprehensive energy policy known as “the National Energy and Mining Policy” in 2017, with the goal of increasing domestic energy utilization, improving energy supply security, and increasing domestic energy market transparency. As a result, this study can provide empirical evidence to support this policy by controlling its necessity and extending the time interval for its application by including additional economic indicators to a conventional environmental-economic model, which includes the trade openness of the country for more accurate results.

Based on the foregoing, this study uses the autoregressive distributed lag (ARDL) time series method to explore the short- and long-run impacts of economic growth, trade openness (abbreviated as TO in the text and *EX* in the model), and energy consumption diversification (i.e., primary energy consumption, coal consumption, and hydroelectricity consumption) on environmental degradation (i.e., CO_2_ emissions and *EF*) from 1971 to 2015 in Turkey. The validity of the EKC hypothesis in the given period is important for future renewable energy investment decisions and could help Turkey deal with environmental degradation caused by CO_2_ emissions and their *EF* because the utilization of more renewable energy sources is a powerful strategy for environmentally friendly energy consumption, which could be the most effective way of achieving sustainable economic growth in the near future. The statistical results of the analysis reveal that TO increases CO_2_ emissions in the long-run, while reducing *EF*. Coal consumption increases both CO_2_ emissions, and *EF*. While hydroelectricity consumption reduces CO_2_ emissions, it has no impact on *EF*. In addition, an inverted U-shape relationship is found between environmental degradation and GDP per capita.

Throughout the process of conducting the statistical model, this work contributes to the current literature body in two ways. Firstly, based on what we know best, this is one of the first research paper related to the EKC hypothesis to examine the impact of energy diversification or types of energy consumption (i.e., primary energy consumption, coal consumption, and hydroelectricity consumption) on Turkey’s *EF*. Secondly, in addition to CO_2_ emissions as an indicator of environmental degradation, *EF* is also included in order to obtain more reliable results with the included TO economic indicator, and for both CO_2_ emissions and *EF*, this research confirms the existence of the EKC hypothesis in a developing county, Turkey. Furthermore, the threshold points are found in terms of environmental degradation and economic growth for shedding light on the current energy policies in Turkey. However, data availability on energy diversification is currently limited. Therefore, related research based on the proposed concept with this study is needed and can be useful in near future both for Turkey and developing countries.

The work continues as follows: In “Literature review”, a literature review of studies examining the effects of economic growth, TO, and energy consumption on environmental degradation is included. In “Data, model, and methodology”, the data set, the model, and the methodology used in the study are explained. “Results and discussion” includes empirical findings and discussion. In “Conclusions and policy implications”, the conclusions and policy implications of the study are given.

## Literature review

The deterioration of environmental quality has created a great concern for humans, and this growing concern about environmental issues has increased efforts to gain information on the circumstances that cause environmental degradation (Dinda 2004). Since the 1990s, the economic models for growth and environmental quality have grown its popularity and become a hot topic. The EKC hypothesis proposes defining a mathematical relationship (i.e., an inverted U-shape) between environmental quality and sustainable economic development through economic growth (Dinda 2004). The inverted U-shape mathematical relationship between environmental degradation and economic growth was first reported in the 1990s by Gene and Alan (1991), Panayotou (1993), Shafik and Bandyopadhyay (1992), and after that, this was initially defined by Panayotou (1993) as the EKC hypothesis. The findings of studies on the EKC hypothesis in the latest literature differ from each other. While a few studies confirm the EKC hypothesis (Ben Cheikh et al. 2021, Sapkota and Bastola 2017, Sharif et al. 2020), some others do not confirm (Pata and Aydin 2020, Zoundi 2017). Therefore, the validity of the EKC hypothesis is an issue that continues to be debated today.

It is important to address why/which environmental degradation indicator is included in order to reach more reliable results in the empirical studies of the EKC hypothesis (Dogan et al. 2020). CO_2_ emissions have been used as a variable in econometric models for environmental degradation in the majority of studies testing the EKC hypothesis (Danish et al. 2019; Farooq et al. 2022; Jiang et al. 2021; Malik et al. 2020; Ozgur et al. 2022; Rahman et al. 2022). However, CO_2_ emissions, which are only an air pollution, may not be an adequate indicator of environmental degradation (Solarin and Bello 2018). *EF* is an important environmental degradation indicator because it measures environmental sustainability and is a more comprehensive gauge of environmental degradation than any other pollutant (Caglar et al. 2021; Solarin 2019, Ulucak and Bilgili 2018). As a result, *EF* has recently been included for a similar purpose (Ahmad et al. 2020; Ahmed et al. 2021; Destek et al. 2018, Pata and Aydin 2020, Pata and Isik 2021, Ulucak and Bilgili 2018).

A discussion of studies that focus on the impact of energy consumption, economic growth, and TO on environmental degradation (CO_2_ emissions and *EF*) is carried out in this section. The literature review created depending on the scope of the study is summarized in Table 1, including author/year, period, method, and findings. Furthermore, the abbreviations that are used are explained in Table 1.

**Table 1:** The literature reviews

Author/year	Period	Method	Findings
Sharif et al. (2020)	1965Q1–2017Q4	QARDL	The EKC hypothesis is tested as correct
Ben Cheikh et al. (2021)	1980–2015	PSTR	The EKC hypothesis is tested as correct
Sapkota and Bastola (2017)	1980–2010	Panel fixed and random effect model	The EKC hypothesis is tested as correct
Zoundi (2017)	1980–2012	Panel cointegration, DOLS, GMM, DFE, MG, and PMG	The EKC hypothesis is tested as not correct
Pata and Aydin (2020)	1965–2016	Fourier Bootstrap ARDL, Fourier Toda-Yamamoto causality test	The EKC hypothesis is tested as not correct
Nasir and Ur Rehman (2011)	1972–2008	Johansen cointegration and VECM	TO $\uparrow$ environmental degradation (i.e., CO_2_ emissions)
Li and Haneklaus (2022)	1979–2019	Panel ARDL, FMOLS, and DOLS	A ( +) is found between TO and environmental degradation
Kohler (2013)	1960–2009	ARDL and VECM	A ( −) is found between TO and CO_2_ emissions
Fang et al. (2020)	1990–2016	ARDL	A ( −) is found between TO and CO_2_ emissions
Liu et al. (2022)	1980–2017	ARDL and Toda-Yamamoto test	A ( −) is found between TO and *EF*
Alola et al. (2019)	1997–2014	PMG-ARDL	A ( −) is found between TO and the *EF*
Destek and Sinha (2020)	1980–2014	Panel FMOLS, DOLS, and CCE-MG	A ( +) is found between TO and *EF*
Mikayilov et al. (2019)	1996–2014	TVC	A ( +) is found between TO and the *EF*
Nathaniel and Khan (2020)	1990–2016	Panel cointegration and AMG	A ( −) is found between TO and *EF*
Saboori and Sulaiman (2013)	1980–2009	ARDL, Johansen cointegration, and VECM	Primary energy consumption, coal consumption, natural gas consumption, and electricity consumption $\uparrow$ CO_2_ emissions, while oil consumption $\downarrow$ CO_2_ emissions
Ben Jebli and Ben Youssef (2015)	1980–2009	ARDL and VECM	While NRE $\uparrow$ CO_2_ emissions, RE $\downarrow$ CO_2_ emissions
Bilgili et al. (2016)	1977–2010	Panel FMOLS, and panel DOLS	RE consumption $\downarrow$ CO_2_ emissions
Liu and Bae (2018)	1970–2015	ARDL, Johansen cointegration, and VECM	RE consumption has no impact on CO_2_ emissions
Hanif et al. (2019)	1990–2013	ARDL	Fossil fuel consumption $\uparrow$ CO_2_ emissions
Pata and Caglar (2021)	1980–2016	Augmented ARDL	RE consumption has no impact on both CO_2_ emissions and *EF*
Pata (2018a)	1974–2014	ARDL, Gregory Hansen and Hatemi-J cointegration tests, FMOLS, and CCR	RE consumption has no effect on CO_2_ emissions
Pata (2018b)	1971–2014	ARDL	While coal consumption $\uparrow$ CO_2_ emissions, alternative energy consumption $\downarrow$ CO_2_ emissions

*ARDL* autoregressive distributed lag. *AMG* augmented mean group. *CCE-MG* common correlated effects mean group. *CCR* canonical cointegrating regression. *DFE* dynamic fixed effect. *DOLS* dynamic OLS. *FMOLS* fully modified ordinary least squares. *GMM* general method of moments. *MG* mean group. *PMG* pooled mean group. *PSTR* panel smooth transition regression. *QARDL* quantile autoregressive lagged. *TVC* time-varying coefficient cointegration approach. *VECM* vector error correction model, *RE* renewable energy, *NRE* non-renewable energy, *TO* trade openness

$\uparrow$ increase, $\downarrow$ decrease, ( +) positive relationship, ( −) negative relationship

Gene and Alan (1991) investigated the impact of foreign trade on environmental degradation through three lenses: (1) scale effect; (2) composition effect; and (3) technical effect. Environmental damage caused by increased production as a result of “trade openness” (abbreviated as TO in the text and *EX* in the model) is explained by the scale effect (Cole 2004). The composition effect explains how the production structure changes as income rises, and at this point, environmental degradation is reduced (Dinda 2004). The stage at which environmentally friendly technologies are used in the production structure is known as “technical impact” (Dinda 2004). The impact of TO on environmental degradation is determined by a country’s development level (Liu et al. 2022). Many papers have been written about the impact of TO on environmental degradation (Jiang et al. 2022, Liu et al. 2022, Pata and Caglar 2021, Shahbaz et al. 2017). While Li and Haneklaus (2022), Nasir and Ur Rehman (2011), Nasir et al. (2021), and Vural (2020) discovered a positive relationship between TO and CO_2_ emissions, Al-Mulali et al. (2015), Fang et al. (2020), and Kohler (2013) discovered a negative relationship. While Alola et al. (2019), Destek and Sinha (2020), and Liu et al. (2022) found that TO reduces *EF*; Mikayilov et al. (2019) and Nathaniel and Khan (2020) found that TO increases *EF*.

In studies testing the EKC hypothesis, one of the most commonly used explanatory variables is energy consumption (Yurtkuran 2021). The increase in data diversity has enabled the analysis of the impact of REC and fossil fuel consumption on environmental degradation. The impact of REC and fossil fuel consumption on environmental degradation has become a significant issue in recent years. Most of the studies show that REC reduces environmental degradation and NREC increases it. For instance, Ben Jebli and Ben Youssef (2015) conducted a Granger causality test using the ARDL and vector error correction model (VECM) for Tunisia from 1980 to 2009 and concluded that while renewable energy reduces CO_2_ emissions, non-renewable energy increases CO_2_ emissions. Bilgili et al. (2016) examined the Organization for Economic Co-operation and Development countries using panel data model from 1977 to 2010 and discovered that REC reduces CO_2_ emissions. Hanif et al. (2019) used the ARDL method to examine 15 developing Asian countries from 1990 to 2013, and the study found that fossil fuels increase CO_2_ emissions. Contrary to these studies, some studies have proven that REC is ineffective in preventing environmental degradation. For instance, Liu and Bae (2018) concluded that REC has no effect on CO_2_ emissions using the ARDL bounds test, Johansen co-integration test, and VECM Granger causality test for China from 1970 to 2015. Pata (2018a) used the ARDL bounds tests, Gregory Hansen and Hatemi-J cointegration tests, and CCR and FMOLS estimators found that REC has no effect on CO_2_ emissions in Turkey from 1974 to 2014. Pata and Caglar (2021) used an augmented ARDL approach to study REC in China from 1980 to 2016 and discovered that it has no effect on CO_2_ emissions or *EF*. Awan et al. (2022a) used data from 10 emerging countries from 1996 to 2015 and applied a model of moment quantile regression and suggested that encouraging renewable energy use is important to mitigate CO_2_ emissions.

Furthermore, the link between environmental degradation and energy consumption has been the subject of many studies (Mrabet and Alsamara 2017, Shahbaz et al. 2014; Wen et al. 2021). Several studies examining the relationships between energy and environmental degradation did not include different types of energy consumption (Agboola et al. 2021; Kahouli et al. 2022; Liu et al. 2022; Mujtaba et al. 2022; Rahman et al. 2021). Nonetheless, the diversity of energy consumption has different effects on environmental degradation (Saboori and Sulaiman 2013). As a result, it is essential to include energy consumption diversity in the analysis. Few studies in the literature have focused on this, particularly on hydroelectricity consumption, (see Bandyopadhyay et al. (2022) for India, Jahanger et al. (2022) for Malaysia, and Tiwari et al. (2022) for Brazil and China). For instance, Saboori and Sulaiman (2013) used the ARDL bounds test and the Johansen-Juselius cointegration test for Malaysia from 1980 to 2008 and found that while primary energy consumption, coal consumption, natural gas consumption, and electrical energy consumption all increase CO_2_ emissions, oil consumption decreases CO_2_ emissions. Pata (2018b) conducted the ARDL bounds test for Turkey, and while he found a positive relationship between coal consumption and CO_2_ emissions, he found a negative relationship between alternative energy consumption and CO_2_ emissions. These studies have proven that energy diversity causes different effects on environmental degradation. However, while doing this, they did not consider *EF* as an indicator of environmental degradation. There has been no research into the impact of energy consumption types on *EF*, particularly in Turkey. Unlike previous studies, this study aims to fill a gap in the literature by analyzing energy consumption diversity (primary energy consumption, coal consumption, and hydroelectric consumption) as well as *EF*.

## Data, model, and methodology

### Data and model

This study investigates the factors affecting environmental degradation in Turkey, a developing economy. In line with the purpose of the study, the EKC hypothesis is used for the economic model. The EKC hypothesis assumes an inverted U-shape relationship between economic growth and environmental degradation. As a result, real GDP per capita (*Y*) and the square of real GDP per capita (*Y*^2^) are used as the main economic indicators in the model. In order to perform a more comprehensive and reliable analysis, both CO_2_ emissions and *EF* variables are used as indicators of environmental degradation. Energy consumption, a crucial component of Turkey’s economic growth, has a significant importance on environmental degradation. Thereby, it is thought necessary to include energy consumption in the model. Energy diversification, on the other hand, can have varying effects on environmental degradation. As a result, the model incorporates energy diversification (i.e., primary energy consumption, coal consumption, and hydroelectric consumption). Because only limited time series data on renewable energy types were available at the time of this study, only hydroelectric consumption was considered as renewable energy use.

Table 2 shows the variables, their explanations, their units, and their sources. Data for CO_2_ emissions, *Y*, and *EX* data are from the World Development Indicators (WDI 2022); data for *EF* is from the Global Footprint Network (Network 2022); and data for *PEC*, *CC*, and *HEC* are from the British Petroleum (BP 2022). When the conditions of ${\beta }_{1}>0$ and ${\beta }_{2}<0$ for Eqs. (1)–(3) and the conditions of ${\alpha }_{1}>0$ and ${\alpha }_{2}<0$ for Eqs. (4)–(6) are supplied, it means that the EKC hypothesis is valid. For both environmental degradation indicators, the threshold value (i.e., turning point) is calculated as ${Y=-\beta }_{1}/{2\beta }_{2}$ (for CO_2_ emissions), $Y={-\alpha }_{1}/{2\alpha }_{2}$ (for ecological footprint), and exp(*Y*). It is expected that the primary energy consumption and coal consumption coefficients will be ( +), while the *HEC* coefficient will be ( −). The TO coefficient can be either ( −) or ( +), depending on the level of the economic growth and development level of the country.

**Table 2:** The variables, explanations, and sources

Variable	Explanation	Source
CO_2_	CO_2_ emissions (per capita metric tons)	World Development Indicators
*EF*	Ecological footprint (global hectares per person)	Global Footprint Network
*Y*	GDP per capita (constant 2015 US $)	World Development Indicators
*Y*^2^	GDP per capita square (constant 2015 US $)	World Development Indicators
*EX*	Goods and services export divided by GDP	World Development Indicators
*PEC*	Primary energy consumption (per capita)	British Petroleum Statistical Review of World Energy 2021
*CC*	Coal consumption (per capita)	British Petroleum Statistical Review of World Energy 2021
*HEC*	Hydroelectricity consumption (per capita)	British Petroleum Statistical Review of World Energy 2021

In line with the above-mentioned explanations, log-linear quadratic models created for testing the validity of the EKC hypothesis are shown with Eqs. (1)–(6). In these equations, CO_2_, *EF*, *Y*, *Y*^2^, *EX*, *PEC*, *CC*, and *HEC* represent CO_2_ emissions per capita, *EF* per capita, real GDP per capita, real GDP squared per capita, trade openness, primary energy consumption per capita, coal consumption per capita, and hydroelectricity consumption per capita, respectively.

1 $${\mathrm{lnCO}}_{2t}= {\beta }_{0}+{\beta }_{1}{\mathrm{lnY}}_{t}+{\beta }_{2}({{\mathrm{ln}Y}_{t})}^{2}+{\beta }_{3}{\mathrm{ln}EX}_{t}+{\beta }_{4}{\mathrm{ln}PEC}_{t}+ {\varepsilon }_{t}$$

2 $${\mathrm{lnCO}}_{2t}= {\beta }_{0}+{\beta }_{1}{\mathrm{ln}Y}_{t}+{\beta }_{2}({{\mathrm{ln}Y}_{t})}^{2}+{\beta }_{3}{\mathrm{ln}EX}_{t}+{\beta }_{4}{\mathrm{ln}CC}_{t}+ {\varepsilon }_{t}$$

3 $${\mathrm{lnCO}}_{2t}= {\beta }_{0}+{\beta }_{1}{\mathrm{ln}Y}_{t}+{\beta }_{2}({{\mathrm{ln}Y}_{t})}^{2}+{\beta }_{3}{\mathrm{ln}EX}_{t}+{\beta }_{4}{\mathrm{ln}HEC}_{t}+ {\varepsilon }_{t}$$

4 $${\mathrm{ln}EF}_{t}= {\alpha }_{0}+{\alpha }_{1}{\mathrm{ln}Y}_{t}+{\alpha }_{2}({{\mathrm{ln}Y}_{t})}^{2}+{\alpha }_{3}{\mathrm{ln}EX}_{t}+{\alpha }_{4}{\mathrm{ln}PEC}_{t}+ {\varepsilon }_{t}$$

5 $${\mathrm{ln}EF}_{t}= {\alpha }_{0}+{\alpha }_{1}{\mathrm{ln}Y}_{t}+{\alpha }_{2}({{\mathrm{ln}Y}_{t})}^{2}+{\alpha }_{3}{\mathrm{ln}EX}_{t}+{\alpha }_{4}{\mathrm{ln}CC}_{t}+ {\varepsilon }_{t}$$

6 $${\mathrm{ln}EF}_{t}= {\alpha }_{0}+{\alpha }_{1}{\mathrm{ln}Y}_{t}+{\alpha }_{2}({{\mathrm{lnY}}_{t})}^{2}+{\alpha }_{3}{\mathrm{ln}EX}_{t}+{\alpha }_{4}{\mathrm{ln}HEC}_{t}+ {\varepsilon }_{t}$$

### The methodology

For the methodology, the autoregressive distributed lag (ARDL) bounds test approach is used to investigate the relationship between environmental degradation, economic growth, trade openness, primary energy consumption, coal consumption, and hydroelectric consumption in Turkey. The ARDL bounds test developed by Pesaran et al. (2001) has three important advantages. First, the ARDL bounds test allows variables to have different degrees of integration. In case the variables are “zero order of integration” (*I*(0)) or “one order of integration” (*I*(1)), cointegration test can be conducted. However, none of the variables should have a degree of integration of “two order of integration” (*I*(2)). Second, unlike traditional cointegration tests, the ARDL bounds test is a suitable approach for small samples. Third, with the ARDL model, both the short- and long-run effects of independent variables on the dependent variable can be considered. Because of these advantages, the long- and short-run relationship between the variables is investigated with the ARDL bounds test. Equation (7) depicts the ARDL bounds test model developed to investigate the cointegration relationship between the variables.

7 $$\Delta {\mathrm{ln}ED}_{t}={\beta }_{0}+\sum_{i=1}^{m}{\beta }_{1i}{\Delta \mathrm{ln}ED}_{t-i}+\sum_{i=0}^{m}{\beta }_{2i}{\Delta \mathrm{ln}Y}_{t-i}+\sum_{i=0}^{m}{\beta }_{3i}{\Delta \mathrm{ln}Y}_{t-i}^{2}+\sum_{i=0}^{m}{\beta }_{4i}{\Delta \mathrm{ln}EX}_{t-i}+\sum_{i=0}^{m}{\beta }_{5i}{\Delta \mathrm{ln}PEC}_{t-i}+\sum_{i=0}^{m}{\beta }_{6i}{\Delta \mathrm{ln}CC}_{t-i}+\sum_{i=0}^{m}{\beta }_{7i}{\Delta \mathrm{ln}HEC}_{t-i}+{\alpha }_{1}{\mathrm{ln}ED}_{t-1}+{\alpha }_{2}{\mathrm{ln}Y}_{t-1}+{\alpha }_{3}{\mathrm{ln}Y}_{t-1}^{2}+{\alpha }_{4}{\mathrm{ln}EX}_{t-1}+{\alpha }_{5}{\mathrm{ln}PEC}_{t-1}+{\alpha }_{6}{\mathrm{ln}CC}_{t-1}+{\alpha }_{7}{\mathrm{ln}HEC}_{t-1}+{e}_{t}$$

In Eq. (7), *ED* refers to environmental degradation (i.e., CO_2_ emissions and *EF*), the constant term is *β*_0_; ∆ refers to the difference operator; *e*_*t*_ refers to the error term; *β*_1_, …, *β*_7_ refers to the short-run coefficients; and *α*_1_, …, *α*_7_ refer to the long-run coefficients.

The H_0_ or “0” hypothesis indicates that there is no cointegration between variables; the alternative hypothesis H_1_ means that there is cointegration between them. If the *F* statistical value obtained from the bounds test is above the upper critical value, the H_0_ is not accepted, and a long-run relationship can be confirmed. The established error correction model (ECM) to estimate the short-run coefficients is given in Eq. (8) as follows:

8 $$\Delta {\mathrm{ln}ED}_{t}={\delta }_{0}+\sum_{i=1}^{n}{\delta }_{1i}{\Delta \mathrm{ln}ED}_{t-i}+\sum_{i=0}^{n}{\delta }_{2i}{\Delta \mathrm{ln}Y}_{t-i}+\sum_{i=0}^{n}{\delta }_{3i}{\Delta \mathrm{ln}Y}_{t-i}^{2}+\sum_{i=0}^{n}{\delta }_{4i}{\Delta \mathrm{ln}EX}_{t-i}+\sum_{i=0}^{n}{\delta }_{5i}{\Delta \mathrm{ln}PEC}_{t-i}+\sum_{i=0}^{n}{\delta }_{6i}{\Delta \mathrm{ln}CC}_{t-i}+\sum_{i=0}^{n}{\delta }_{7i}{\Delta \mathrm{ln}HEC}_{t-i}+{\gamma ECT}_{t-1}+ {e}_{t}$$

**Fig. 2:**
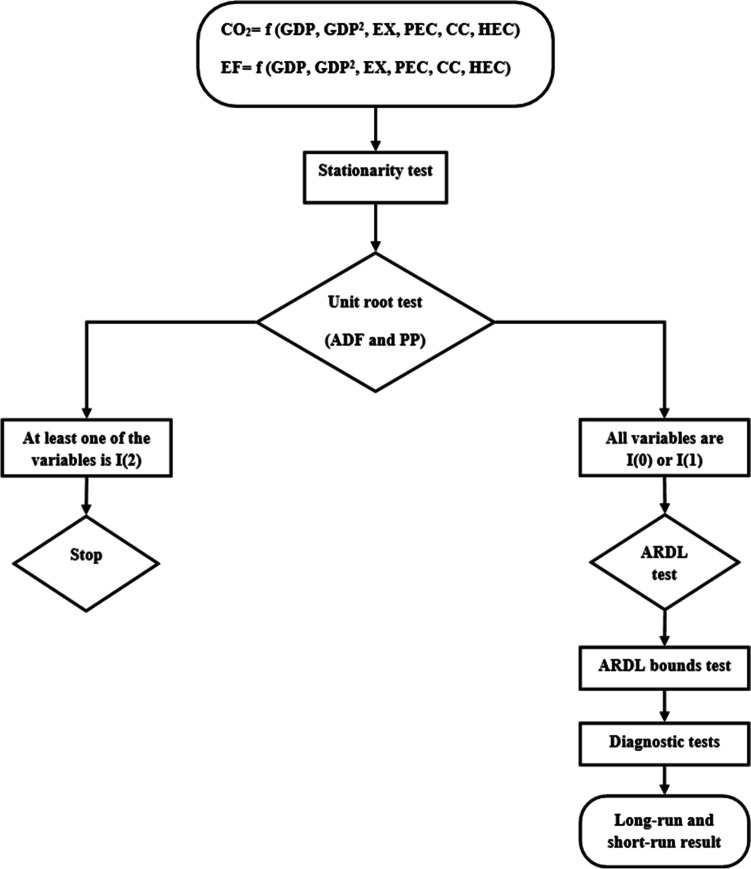
Flow chart of the applied methodology

In Eq. (8), *δ*_0_ refers to the constant term; ∆ refers to the difference operator; *e*_*t*_ refers to the error term; *δ*_1_, …, *δ*_7_ refer to the short-run coefficients. The error correction term (${ECT}_{t-1}$) indicates how long it will take for short-run shocks to reach long-run equilibrium, and *γ* should be ( −) and statistically significant. In order to test the validity of the statistical findings obtained as a result of the ARDL model, diagnostic tests are included. Furthermore, cumulative sum of recursive residuals (CUSUM) and cumulative sum of squares of recursive residuals (CUSUMSQ) tests are conducted to determine if the coefficients of all models are stable. Figure 2 presents the flow chart of the used methodology.

## Results and discussion

Firstly, the augmented Dickey Fuller (ADF) and Phillips Perron (PP) unit root tests are used to determine the variables’ stationarity level. Table 3 displays the results of the ADF and PP unit root tests. According to the ADF unit root test results, all series have unit root at the level, but not after the first difference is taken. All series except *HEC* have unit root at the level, according to the PP unit root test results. Nonetheless, when the first difference is taken, it is seen that they do not include a unit root. In accordance with the results of the ADF and PP unit root tests, none of the variables are *I*(2). Therefore, it can be agreed that the ARDL model is a suitable approach.

**Table 3:** The ADF and PP unit root test results

Variables	ADF		PP	
	Level	First difference	Level	First difference
CO_2_	− 1.4403	− 6.7687***	− 1.5825	− 7.5309***
*EF*	− 0.8035	− 10.2840***	− 0.9680	− 13.9432***
*Y*	0.5807	− 6.3189***	0.6325	− 6.3198***
*Y*^2^	0.7657	− 6.2600***	0.8330	− 6.2617***
*EX*	− 1.3773	− 5.8942***	− 1.4030	− 5.8942***
*PEC*	− 1.7899	− 6.7635***	− 1.9757	− 6.7650***
*CC*	− 1.2094	− 7.0461***	− 1.2094	− 7.0461***
*HEC*	− 2.3856	− 7.3816***	− 3.0572**	− 7.6926***

The lag lengths for the ADF test are prepared according to the Schwarz information criterion. The lag lengths for the PP unit root test are prepared according to the Newey-West information criterion

^***^, **, * denote significance at the 1%, 5%, and 10% levels, respectively

Table 4 shows the ARDL bounds test results for six models with the appropriate lag length, *F* statistics, and integration degree. As a result of the *F* value being above the upper limit critical values, the long-run cointegration relationship can be determined in all models.

**Table 4:** The ARDL bounds test results

Models	Lag length	*F*-statistics	*I*(0)	*I*(1)
Model 1	(1,0,3,1,3)	6.04117	2.893	4
Model 2	(2,0,3,0,2)	6.4292	2.893	4
Model 3	(1,0,0,0,0)	18.6621	2.85	3.905
Model 4	(1,1,0,0,1)	20.6835	2.85	3.905
Model 5	(2,1,0,3,0)	9.5424	2.893	4
Model 6	(1,1,0,0,0)	16.1058	2.85	3.905

*I*(0) and *I*(1) values in the table are written according to the 5% significance level

Table 5 displays the diagnostic test results for the models. According to the results in Table 5, the Breusch-Pagan-Godfrey (BPG) test has no problem with time-varying variance; the Lagrange Multiplier (LM) test has no problem with autocorrelation; the error terms have a normal distribution with the Jarque–Bera (JB) test; and the Ramsey RESET test has no problem with specification.

**Table 5:** The diagnostic test results

Models	BPG	LM	JB	RAMSEY	*R*^2^
Model 1	17.0462 (0.1479)	0.4782 (0.7873)	0.3561 (0.8368)	0.0016 (0.9676)	0.9940
Model 2	10.4716 (0.4885)	2.0578 (0.3574)	3.0429 (0.2183)	0.1960 (0.6612)	0.9959
Model 3	2.8768 (0.7190)	0.3658 (0.8328)	0.3458 (0.8411)	0.4072 (0.5273)	0.9925
Model 4	4.3374 (0.7402)	0.9587 (0.6192)	5.4138 (0.0667)	1.3752 (0.2488)	0.9801
Model 5	11.7131 (0.3047)	1.4523 (0.4838)	4.0080 (0.1347)	1.0164 (0.3214)	0.9836
Model 6	6.3608 (0.3840)	0.4111 (0.8142)	1.4065 (0.4949)	0.6113 (0.4394)	0.9755

The values in parentheses refer the probability value

Table 6 shows the estimated ARDL long-run, and Table 7 shows the estimated ARDL short-run results for CO_2_-emissions models. Long-run results show that the GDP coefficient is ( +) and the GDP squared coefficient is ( −). GDP and CO_2_ emissions have an inverted U-shape, indicating that the EKC hypothesis is correct. The price thresholds are $18,704, $16,361, and $13,571. Nonetheless, these thresholds are higher than GDP per capita for the time periods studied (i.e., the maximum value of GDP per capita for the period is $11,006). This finding is comparable to those of references (Bölük and Mert 2015, Pata 2018a, b). The rapid population and economic growth in the last 20 years in Turkey both increased the energy demand and import dependency. As a solution, Turkey followed a strategy of restructuring its energy system, including lower energy prices for consumers, rationalizing energy demand growth, and decreasing the pace of import growth (IEA 2021). These energy policies should be followed with patience. According to the study’s findings, after a certain level of income, Turkey will be able to reduce environmental degradation issues and achieve sustainable economic growth.

**Table 6:** The ARDL long-run estimation results for CO_2_ emissions

Variables	Model 1		Model 2		Model 3	
	**Coefficient**	***t* statistics**	**Coefficient**	***t* statistics**	**Coefficient**	***t* statistics**
*Y*	7.9066***	2.9425	8.3152***	7.8309	12.1554***	6.9834
*Y*^2^	− 0.4019***	− 2.8562	− 0.4285***	− 7.2898	− 0.6387***	− 6.5316
*EX*	0.0497**	2.0952	0.0014	0.1126	0.0525***	2.7877
*PEC*	0.0650	0.4503				
*CC*			0.1896***	3.9278		
*HEC*					− 0.0498**	− 2.0265

^***^, **, * denote significance at the 1%, 5%, and 10% levels, respectively

**Table 7:** The ARDL short-run estimation results for CO_2_ emissions

**Variables**	**Model 1**		**Model 2**		**Model 3**	
	**Coefficient**	***t* statistics**	**Coefficient**	***t* statistics**	**Coefficient**	***t* statistics**
CO_2_ (− 1)			0.3500***	2.8915		
*Y*^2^	− 0.4736***	− 6.3524	− 0.4850***	− 6.3408		
*Y*^2^ (− 1)	− 0.0306***	− 3.4728	− 0.0201***	− 3.1486		
*Y*^2^ (− 2)	− 0.0236***	− 2.8490	− 0.0100**	− 2.2266		
*EX*	0.0113	0.4633				
*PEC*	0.5394***	4.7975				
*PEC* (− 1)	0.4450***	3.7760				
*PEC* (− 2)	0.2173*	1.8984				
*CC*			0.3379***	6.9402		
*CC* (− 1)			− 0.1023	− 1.5755		
*ECT* (− 1)	− 1.0762***	− 6.5192	− 1.0824***	− 6.7085	− 0.8045***	− 11.2563

^***^, **, * denote significance at the 1%, 5%, and 10% levels, respectively

In the long-run, the TO coefficient is ( +) and significant. A 1% increase in export rate increases CO_2_ emissions by 0.0497% for model 1 and 0.0525% for model 3 (note that model 2 gives insignificant result). The finding that TO increases CO_2_ emissions is consistent with the findings of Halicioglu (2009), Ozatac et al. (2017), and Pata (2019). The export structure of Turkey is based on low-technology products (TÜİK 2022b). This could explain the rise in CO_2_ emissions caused by increased exports. Furthermore, the increase in environmental degradation as a result of TO suggests that the scale effect is dominant in Turkey. On the other hand, Turkey prioritized the security of energy supply because of its heavy dependence on gas and oil imports. In the last 10 years, Turkey has made a significant progress toward liberalization of energy markets, with increased transparency and pricing predictability (IEA 2021). In line with the finding of this study, it will be better to continue with this policy.

While a 1% increase in primary energy consumption increases CO_2_ emissions by 0.5394% in the short-run, it has no effect in the long-run. The intuition behind this finding can be explained by the fact that Turkey’s energy mix has undergone significant diversification in the last 10 years. To this end, the energy policy covers the production of domestic oil and gas exploration, the diversification of oil and gas supply sources, associated infrastructure, and the reduction of energy consumption through increased energy efficiency. Furthermore, the coefficient of coal consumption is ( +) and significant both in the short-run and in the long-run. A 1% increase in coal consumption increases CO_2_ emissions by 0.3379% in the short-run and 0.1896% in the long-run. According to the findings, the effect of coal consumption on CO_2_ emissions is greater in the short-run than in the long-run.

On the other hand, the hydroelectricity consumption coefficient is ( −) and significant in the long-run. An increase of 1% in hydroelectricity consumption reduces CO_2_ emissions by 0.0498%. According to the findings, fossil fuel consumption increases CO_2_ emissions, while REC decreases CO_2_ emissions. This finding is comparable to those of Bulut (2017) and Karaaslan and Çamkaya (2022). Here, it is worth mentioning that the notable growth in Turkey’s energy generation (not only in hydro but also in wind and solar energy) led to renewable electricity generation, and this is a very powerful strategy for mitigating global warming through a lower amount of CO_2_ emissions.

Technically speaking, the values of the error correction coefficients are statistically significant and ( −). As a result, it is possible to say that short-run deviations can be eliminated within a year. The short-run estimation results are shown in Table 7. The coefficient of error correction term is significant and ( −) in all models, as expected.

Table 8 displays the estimated ARDL long-run, and Table 9 displays the short-run results for models with *EF* as the dependent variable. According to the long-run results, the GDP coefficient is ( +) and the GDP square is ( −). As a result, the EKC hypothesis is validated, confirming an inverted U-shape relationship between *EF* and GDP. The calculated threshold points are $11,824, $11,821, and $15,476, respectively. Nevertheless, these threshold points are higher than GDP per capita for the periods considered (i.e., the maximum value of GDP per capita for the periods considered is $11,006). This finding is consistent with the findings obtained for CO_2_ emissions. The TO coefficient is both ( −) and significant both in the long-run and the short-run. A 1% increase in export rates reduces *EF* in the long-run by 0.0916%, 0.1407%, and 0.0629%, and in the short-run by 0.0777%. This finding is consistent with the Liu et al.’s (2022) and Nathaniel and Khan’s 2020) findings of a ( −) relationship between *EF* and TO. Table 6 shows that TO increases CO_2_ emissions, whereas Table 8 shows that TO decreases *EF*. The findings show that the impact of TO on environmental degradation varies depending on the environmental degradation indicator used in Turkey.

**Table 8:** The ARDL long-run estimation results for the *EF*

Variables	Model 4		Model 5		Model 6	
	**Coefficient**	***t* statistics**	**Coefficient**	***t* statistics**	**Coefficient**	***t* statistics**
*Y*	4.2876*	1.9191	8.0704***	7.4392	6.0545***	4.0562
*Y*^2^	− 0.2286*	− 1.9345	− 0.4303***	− 7.0711	− 0.3138***	− 3.7385
*EX*	− 0.0916***	− 3.9779	− 0.1407***	− 6.3897	− 0.0629***	− 3.8407
*PEC*	0.1983*	1.7334				
*CC*			0.1784***	4.2624		
*HEC*					0.0276	1.4052

^***^, **, * denote significance at the 1%, 5%, and 10% levels, respectively

**Table 9:** The ARDL short-run estimation results for the *EF*

Variables	Model 4		Model 5		Model 6	
	**Coefficient**	***t* statistics**	**Coefficient**	***t* statistics**	**Coefficient**	***t* statistics**
*EF* (− 1)			0.1430	1.6002		
*Y*	4.2735***	14.3907	10.7425***	8.9807	6.3134***	12.3416
*EX*			− 0.0777***	− 3.6293		
*EX* (− 1)			0.1034***	3.5405		
*EX* (− 2)			0.0731***	3.6359		
*PEC*	0.4905***	5.1399				
*ECT* (− 1)	− 0.9008***	− 11.8885	− 1.2900***	− 8.1541	− 0.9616***	− 10.4734

^***^, **, * denote significance at the 1%, 5%, and 10% levels, respectively

The primary energy consumption coefficient is both ( +) and significant both in the short- and long-run. A 1% increase in primary energy consumption raises *EF* by 0.4905% in the short-run, but by 0.1983% in the long-run. According to this finding, the short-run impact of primary energy consumption on *EF* is greater than the long-run impact. Natural gas, oil, and, coal dominate Turkey’s primary energy consumption (Agency 2021). This finding emphasizes the ( −) impact of fossil fuel consumption on *EF*. The coal consumption coefficient is both ( +) and statistically significant. Numerically, a 1% increase in coal consumption results in a 0.1784% increase in *EF*. These findings are similar to those of references (Destek and Sinha 2020, Sharif et al. 2020). Furthermore, Awan et al. (2022c) shows that REC and NREC have significantly ( −) and ( +) effects on environmental degradation via CO_2_ emissions for 107 countries from 1996 to 2014.

**Fig. 3:**
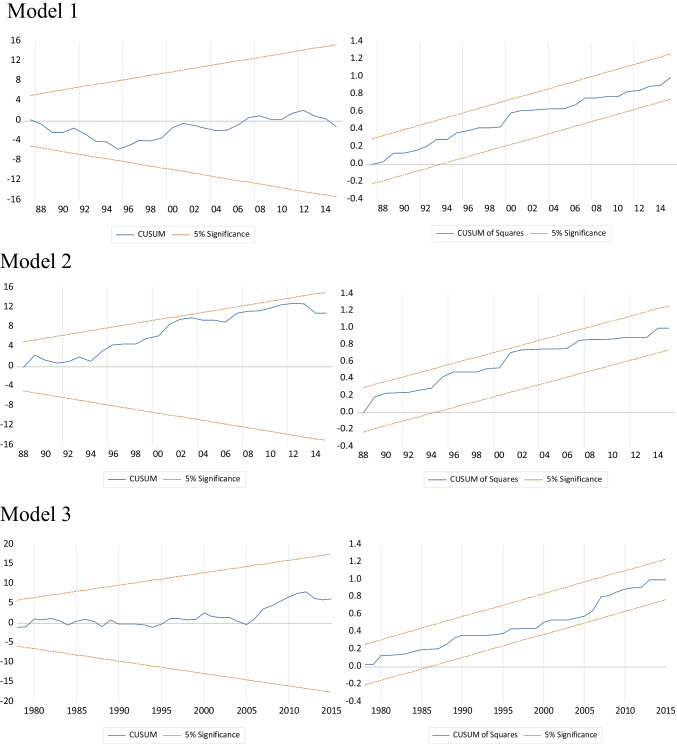

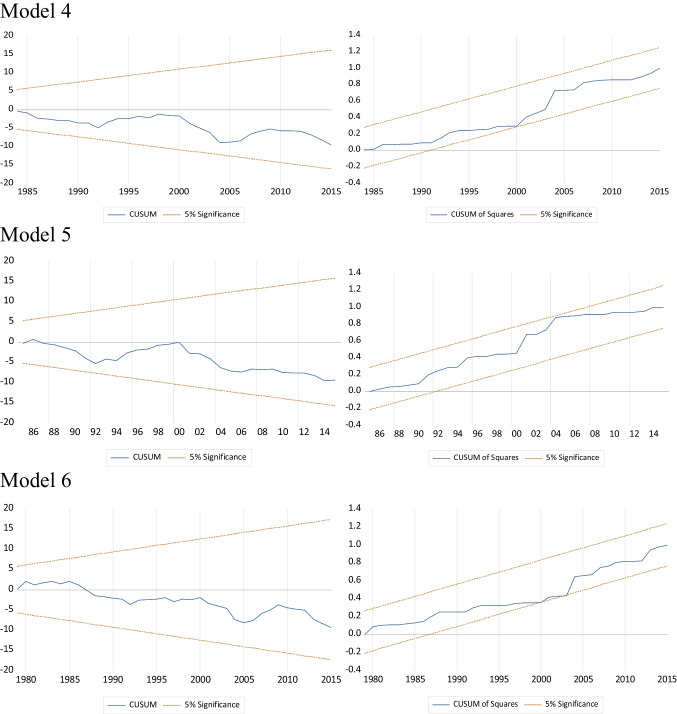
CUSUM VE CUSUMSQ tests

In terms of REC, Tiwari et al. (2022) discovered contradictory findings on hydropower consumption in China and Brazil. Their findings support the ( +) effects of hydropower consumption on *EF* in China, but they were insignificant in Brazil, which is consistent with the findings of this study in Turkey. Nonetheless, in contrast to those research studies, this study supports hydropower consumption’s ( +) effects on CO_2_ emissions. However, findings indicate that hydroelectricity consumption has no effect on *EF*. This finding suggests that hydroelectricity resources in Turkey are not being used effectively to reduce *EF*, which could be a good research topic for academics. The values of the error correction coefficients are statistically significant and ( −). As a result, it is possible to say that short-run deviations can be eliminated within a year. Table 9 shows the short-run estimation results. According to the results, the error correction coefficient terms in all models are ( −) and significant as expected. The coefficients of all models are stable according to the CUSUM and CUSUMSQ tests, as shown in Fig. 3.

Aside from the model and variables used in this study, the linear and non-linear effects of urbanization and technological innovation in the conceptual framework of the EKC hypothesis may help reduce CO_2_ emissions through increased use of public transportation, non-motorized vehicles, and urbanization ((Awan et al. 2022b), (Awan et al. 2022c), (Awan et al. 2022d)). These methods and approaches are currently found in small numbers in the literature, but the researchers in less-developed, developing, and developed countries must increase the number.

If the necessary data for energy diversification is available, the economic model of this study can be applied to any countries. Countries’ institutions should be required to collect and share this data with the public. At this stage, developed countries have an advantage. Renewable energy sources are becoming more viable in developed countries, and data observation is becoming more feasible. Furthermore, the institution quality is high, which improves transparency in the energy markets. In developing countries, the situation is different. Although they may have potential renewable energy sources, they may be far from using their domestic potential, as in the case of Turkey. Nonetheless, the increase in the number of quantitative works, likewise this study, will inform the decision-makers, and as a result, environmentally friendly energy investments (i.e., for renewable energy sources) could be higher.

## Conclusions and policy implications

The goal of this work is to test the existence of the environmental Kuznets curve hypothesis in Turkey from 1971 to 2015 for both carbon dioxide emissions and ecological footprint through energy diversification. The autoregressive distributed lag (ARDL) bounds test is used to investigate the effects of primary energy consumption, coal consumption, hydroelectricity consumption, gross domestic product, and trade openness on environmental degradation. The findings show that the environmental Kuznets curve hypothesis is valid for both carbon dioxide emissions with the threshold points of $18,704, $16,361, and $13,571 for models 1–3, respectively, and ecological footprint with the threshold points $11,824, $11,821, and $15,476 for models 4–6, respectively, both of which are higher than the maximum value of gross domestic product per capita in the periods included in the analysis with the threshold of $11,006.

Specifically, in the long-run, trade openness increases carbon dioxide emissions and reduces ecological footprint. While primary energy consumption increases carbon dioxide emissions in the short-run, it has no effect in the long-run. Primary energy consumption increases ecological footprint both in the short- and long-run. Coal consumption increases carbon dioxide emissions as well as ecological footprint. While hydroelectricity consumption reduces carbon dioxide emissions, it has no effect on ecological footprint to reduce environmental degradation and ensure sustainable economic growth and development; the proportion of total energy consumption dedicated to fossil fuels should be reduced, while the proportion dedicated to renewable energy should be increased. Furthermore, increasing the proportion of renewable energy consumption in total energy consumption may benefit Turkey’s economic growth by reducing the country’s reliance on foreign energy.

The findings of this study shed light on various policy implications for sustainable economic growth and environmental degradation through energy diversification in Turkey. First, the validity of inverted U-shape relationship between ecological footprint and economic growth recommends that Turkey’s policy to reduce ecological degradation should continue for sustainable economic growth in the cleanest possible energy sector and green environment. In this context, Turkey has a promising future there with a heating sector and electricity sector that uses renewable energy sources. Furthermore, the first nuclear power facility, which is planned to be commissioned in 2023, will diversify Turkey’s fuel mix. Second, Turkey should be aware of the importance of a low-level ecological footprint for a sustainable economic growth, and its industrial policy should be infrastructured around a clean energy transition based on energy diversification. Third, given that the relationship between CO_2_ emissions and economic growth validates an inverted U-shape curve in Turkey, the economic growth level in Turkey’s economy seems sufficient to reach acceptable reductions in CO_2_ emissions after the indicated income levels. It is recommended that Turkey follows a strategic energy policy that achieves the tradeoff between economic growth and environmental degradation. This energy strategy should be encouraged by government subsidies to reduce CO_2_ emissions. To that end, advances in energy storage, electric vehicles, and digital technologies may be the strategic paths to take in the near future. Furthermore, for sustainable economic growth in a green environment, Turkey should seek cost-effective and domestic opportunities to meet its energy demand through energy diversification.

As a result, Turkey is dependent on foreign sources for energy consumption at a rate of approximately 74%, ensuring energy supply security is a very important issue. With its National Energy and Mining Policy in 2017, Turkey focused on energy supply security, domestic energy production, and increasing renewable energy resources. In recent years, the share of renewable energy consumption in Turkey has increased. However, the consumption share of hydroelectricity energy in primary energy consumption has decreased. There is no consensus in the literature on the impact of hydroelectricity consumption on environmental degradation. However, according to the findings of this study, hydroelectricity consumption in Turkey reduces carbon dioxide emissions. Based on the findings of this study, it is recommended to increase the share of hydroelectricity consumption in total energy consumption in Turkey. At the same time, Turkey aims to decrease its energy imports by increasing its domestic coal production. However, the findings of this study show that coal consumption increases environmental degradation (carbon dioxide emissions and ecological footprint). For this reason, it is recommended to reduce the share of coal consumption in total energy consumption and to develop cleaner coal technologies.

As a limitation of the research, it is worth noting that there were insufficient time series data on renewable energy types in the official records. Therefore, only hydroelectricity consumption was considered a renewable energy source. If a sufficient data set is provided, modeling of different types of renewable energy can be provided separately in future studies. Nevertheless, this analysis can serve as an econometric time series model for other developing countries.

## Data Availability

Not applicable.
